# Comparison of Anterior Corneal Aberrometry, Keratometry and Pupil Size with Scheimpflug Tomography and Ray Tracing Aberrometer

**DOI:** 10.3390/vision6010018

**Published:** 2022-03-18

**Authors:** Zahra Ashena, Sean Gallagher, Hasan Naveed, David J. Spalton, Mayank A. Nanavaty

**Affiliations:** 1 Sussex Eye Hospital, University Hospitals Sussex NHS Foundation Trust, Brighton BN2 5BF, UK; zashena@nhs.net (Z.A.); s.gallagher@liverpool.ac.uk (S.G.); hasan.naveed@nhs.net (H.N.); 2 Maidstone Hospital, Maidstone and Tunbridge Wells NHS Trust, Maidstone TN2 4QJ, UK; 3 Life Science and Medicine Department, Kings College, London WC2R 2LS, UK; dspalton@hotmail.com

**Keywords:** aberrometry, Scheimpflug, tomography, ray tracing, keratometry

## Abstract

This study aimed to assess the anterior corneal wavefront aberrations, keratometry, astigmatism vectors and pupil size between Pentacam HR^®^ (Oculus Optikgeraete GmbH, Wetzlar, Germany) and iTrace^®^ (Tracey Technologies Corp., Houston, TX, USA). In this observational study, 100 eyes (50 healthy volunteers) were scanned in mesopic light condition with a Pentacam HR^®^ and iTrace^®^. Anterior corneal aberrations (spherical aberration (Z40), vertical coma (Z3 − 1), horizontal coma (Z3 + 1)), keratometry in the flattest (K1) and steepest meridian (K2), mean astigmatism, astigmatic vectors (J0 and J45), and pupil size were measured. We found a significant difference in Z40 (Pentacam^®^: +0.30 ± 0.11 µm and iTrace^®^: −0.03 µm ± 0.05 µm; *p* < 0.01) with no correlation between the devices (r = −0.12, *p* = 0.22). The devices were in complete agreement for Z3 − 1 (*p* = 0.78) and Z3 + 1 (*p* = 0.39), with significant correlation between the machines (r = −0.38, *p* < 0.01 and r = −0.6, *p* < 0.01). There was no difference in K1, K2 and mean astigmatism. J0 was negative with both devices (against-the-rule astigmatism), but there was no correlation. J45 was negative with the Pentacam HR^®^ (more myopic oblique astigmatism) but significantly correlated between the devices. Pupil size was smaller with Pentacam HR^®^ (*p* < 0.01). In summary, these devices cannot be used interchangeably. Corneal Z40 was significantly different with more negative Z40 with iTrace^®^ compared to Pentacam HR^®^. iTrace^®^ operates with lower illumination, giving larger pupil size than Pentacam HR^®^, which uses intense blue light during measurement. No correlation was found for J0. Pentacam HR^®^ had a trend to record more negative J45 (myopic oblique astigmatism).

## 1. Introduction

Keratometric data can be collected through various topography and tomography methods, including slit-scanning elevation topography, Placido disc-based keratoscopy, Scheimpflug imaging and Optical coherence tomography. Different devices use different technologies to measure the HOA [1]. Pentacam HR^®^ (Oculus Optikgeraete GmbH, Wetzlar, Germany) employs a rotating Scheimpflug principle to obtain the data and is a commonly used corneal tomographic technology in clinical practice [2]. Additionally, Pentacam HR^®^ can produce the measurements of the corneal higher-order aberrations (HOA) by computing the information from the tomography scans. Wavefront aberrometers have also been increasingly popular with the development of wavefront-guided corneal refractive surgery, wavefront-customised intraocular lenses, and aberration-correcting contact lenses in the past years [3]. They provide a measurement of the corneal and total ocular HOA. Three different wavefront measuring technologies are available to measure aberrations: Hartmann-Shack, Tscherning or ray tracing (e.g., iTrace^®^, Tracey Technologies Corp. Houston, TX, USA), and automated retinoscopy [4]. Hartmann-Shack is the wavefront technology that uses one shot to measure a wavefront, which is quick and produces high-resolution evaluations. In comparison, ray tracing uses consecutive measurements completed over several sampling points within milliseconds; thus, eye movement does not affect the measurement. Automated retinoscopy is based on dynamic skiascopy. The retina is scanned with a slit-shaped light beam, and the reflected light is captured by an array of rotating photodetectors over a 360° area.

Pupil size and decentration have important implications for corneal refractive surgery beyond the calculations of keratometry and corneal aberrations [5]. Postoperative visual quality may be affected significantly if the surgery is performed based on centration derived from constricted pupils, but a decentred ablation is expected under low illumination (due to bigger pupils), and this may be significant for wavefront-guided ablation treatments [6]. Aiming for more aberration correction may lead to higher sensitive of the system to decentration. Therefore, tolerance limits to decentration for these treatments may be more affected by these misalignments. Aspheric intraocular lens (IOLs) in cataract surgery may have a similar problem [7], where the centration of the IOLs are usually performed under dilated pupils during cataract surgery. However, any shift in the position of the centre of the pupil after constriction under normal photopic illumination may have implications on postoperative optical quality.

Various devices have been developed for anterior keratometry and corneal aberrometry using one or more technologies. With the evolution of new techniques and instruments, one of the critical questions for clinicians is whether these devices could be used interchangeably. Oculus Pentacam HR^®^ and iTrace^®^ employ different techniques for keratometry and aberrometry. There is a paucity of studies exploring anterior keratometry, astigmatism vectors, and corneal aberrometry between these devices. This study compared the anterior corneal spherical and coma wavefront aberrations, keratometry, astigmatism vectors and pupil size between Pentacam HR^®^ and iTrace^®^.

## 2. Material and Methods

This was a prospective, non-interventional, observational study on healthy volunteers between May and August 2020 at the Sussex Eye Hospital, University Hospitals Sussex NHS Foundation Trust, Brighton, United Kingdom. Informed consent was obtained from all the participants, and the nature of the study was explained before assessments. The study was approved by the Hospital’s Audit department and followed the tenets of the Declaration of Helsinki.

The inclusion criteria were adult volunteers with healthy, clear corneas. Exclusion criteria were the presence of dry eyes or any coexisting ocular surface pathology, contact lens wear, corneal scar, keratoconus or any other corneal pathology, high refractive errors (spherical equivalent > ±3 diopters), high refractive cylinders (>1.5 diopters), previous intraocular or keratorefractive surgery and inability to cooperate for the examination on two instruments.

All participants underwent measurements on Pentacam HR^®^ and iTrace^®^ in mesopic light conditions (20 Lux) by a single observer (SG). The subjects were asked to place their chin on the chinrest and press the forehead against the forehead strap. They were asked to blink twice and then look at the fixation device before each measurement. The two systems automatically obtained multiple images of the cornea within their respective acquisition periods. 

Pentacam utilises the rotating Scheimpflug principle to provide data on the anterior and posterior corneal topography and elevation, pachymetry, anterior chamber parameters, pupil size, and other indices. For Pentacam HR^®^ measurements, subjects were asked to look at the fixation target. The machine automatically commenced its measurements when it detected correct alignment with the corneal apex and achieved optimal focus. Pentacam HR^®^ measurement protocol includes a series of 25 images (1003 × 520 pixels) taken over different meridians with a constant blue light source. The acquisition protocol takes 2 s to complete generates a three-dimensional model of the anterior segment with 138,000 elevation points. All scans were taken in the same room in standardized mesopic light conditions for all participants. It automatically converts the corneal elevation profile into corneal wavefront data using the Zernike polynomials with an expansion up to the 10th order. The Zernike’s terms are defined at a maximum diameter of 6 mm. We used Pentacam software version 1.20r36 in this study.

The iTrace^®^ system uses the principles of ray tracing to measure the total ocular HOA. Similarly, it can also provide keratometry, corneal topography and obtain pupil size parameters. Ray tracing uses a narrow laser beam directed into the eye parallel to the eye’s line of sight through an x–y scanner. For aberrometry, the direction of the rays is determined by a crystal with electrical impulses (AOD or Acoustic Optical Deflector), thus no moving parts. For Topography, the iTrace^®^ takes an image with a CCD camera of the tear film. The camera has no moving parts, and the image is instantaneous. The image is taken on the apex of the cornea; thus, as soon as the reflection of the illuminated cone is detected, the image is taken. The iTrace^®^ makes the corneal aberrometry by computing the data obtained through the Placido Eyesys Vista and the entire eye ray tracing aberrometry. Using the refractive map, a transformation in Zernike polynomials is made, and the corneal aberrometry is obtained. Software version 6.2.0 was used for iTrace^®^ measurements in our study. 

Poor quality scans (with artefacts due to misalignment or blinking) were discarded, and measurements were repeated until the best quality scans were obtained. This means for Pentacam HR^®^ measurements, the measurements were repeated until the quality score (QS) displayed ‘OK’ on the screen. For iTrace^®^, this meant that the scans were repeated, and all the 256 points checked individually. Anterior corneal aberrometry measurements were obtained from the central 6mm for both devices as per the respective properties of the devices. Keratometry data were acquired from the central 3 mm of the cornea.

Power vectors (J0 and J45) were used to analyse astigmatism. Thibos and Horner [8] described this vectorial analysis whereby any refractive or keratometric error can be expressed as a combination of 3 orthogonal components: M, J0, and J45. M is the spherical equivalent (SE) in standard clinical terms and is not relevant for studying astigmatism. The J0 component is the power of a Jackson cross-cylinder with axes at 180° and 90°. The J45 component denotes the power of a Jackson cross-cylinder with its axes at 45° and 135° (oblique astigmatism) [8]. For both devices, the following anterior corneal parameters were obtained: spherical aberration (Z40), vertical coma (Z3 − 1), horizontal coma (Z3 + 1), flattest keratometry (K1), steepest keratometry (K2), mean anterior keratometry (Km), negative cylinder, horizontal and oblique astigmatism vectors (J0 and J45) and pupil size in mesopic condition. Data were recorded on Microsoft™ Excel (Microsoft Inc., Albuquerque, NM, USA). The Kolmogorov–Smirnov method was used to analyse normality of the data. As the data were normally distributed, the paired *t* test was used to compare the data. The Pearson correlation coefficient (r) was used to evaluate each correlation statistically. The Bland–Altman method [9] was used to assess the agreement between the devices which plots the inter-device differences between the measurements (*y*-axis) against their mean (*x*-axis) for the raw data. The 95% limits of agreements (LoA) were defined as the mean ± two standard deviations (SD) of the differences between Pentacam HR^®^ and iTrace^®^ instruments. A P value less than 0.05 was considered statistically significant.

## 3. Results

**Figure 1:**
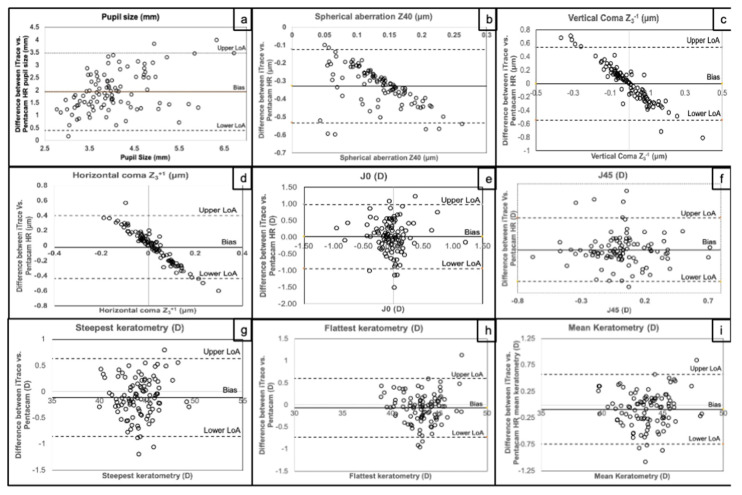
Bland–Altman plot for the difference between iTrace^®^ and Pentacam HR^®^ (**a**) for pupil size, (**b**) for spherical aberration, (**c**) for vertical coma, (**d**) for horizontal coma, (**e**) for J0, (**f**) for J45, (**g**) for steepest keratometry, (**h**) for flattest keratometry, and (**i**) for mean keratometry.

The study comprised 100 eyes (50 individuals) with a mean age of 39.06 ± 11.56 years (range: 21 to 67 years). Pupil size was significantly larger with iTrace^®^ compared to Pentacam HR^®^ and showed a strong positive correlation between the devices (Table 1, Figure 1a). This suggests that Pentacam HR^®^ consistently recorded a smaller pupil size compared to iTrace^®^.

There was a significant difference between the devices (*p* < 0.01) in spherical aberration (Z40), with Pentacam HR^®^ showing a trend towards positive spherical aberration compared to iTrace^®^, which showed more negative spherical aberration, but there was no significant correlation between the devices (Table 1, Figure 1b).

With vertical (Z3 − 1) and horizontal (Z3 + 1) coma aberrations, there was no significant difference between the iTrace^®^ and Pentacam HR^®^, but there was a significant correlation between the two devices (Table 1, Figure 1c,d).

For corneal J0 and J45, there was no significant difference between iTrace^®^ and Pentacam HR^®^. Although J0 was negative with both devices, indicating a trend towards slight against-the-rule astigmatism, there was no correlation between the devices. J45 was only negative with Pentacam HR^®^, indicating more myopic oblique astigmatism. There was a significant correlation between the devices with J45 (Table 1, Figure 1e,f).

Regarding keratometry, there was no significant difference between steepest and flattest keratometry or their meridians, the mean keratometry or mean keratometry meridian or mean keratometric astigmatism. There was a positive and significant correlation between the devices for all these keratometric measurements (Table 1, Figure 1g–i).

## 4. Discussion

Keratometric measurements are crucial for accurate IOL power calculation, refractive surgery and monitoring corneal ectasia. Pentacam HR^®^ has been demonstrated to have high repeatability and reproducibility in various measurements, including anterior keratometry [10]. Similarly, iTrace^®^ also provides precise keratometric measurements with high repeatability and reproducibility [11].

In our study, there was no significant difference between devices for the magnitude and meridian of the steep K, flat K and mean K measurements. They were in complete agreement with a significant positive correlation between the devices (Table 1). Some of the older versions of Scheimpflug scanners (Pentacam^®^, Oculus Optikgeräte GmbH) and Placido-based scanners (Keratron Scout, Optikon 2000 SpA) were not in agreement for keratometry [12,13]. However, good agreement between newer versions of Scheimpflug and Placido-based topographers in anterior keratometric measurements have been reported in other studies [14,15]. Tajbakhsh et al. assessed the above values in 115 healthy candidates using Pentacam HR^®^ and TMS-4^®^ topographer and reported that these apparatuses might be used interchangeably for anterior keratometric measurements [14]. Huang et al. also compared OphthaTop^®^ (Hummel AG, Denzlingen, Germany), a Placido-based corneal topographer with Pentacam HR^®^ [15] and found the devices in complete agreement. 

Regarding corneal astigmatism, we did not notice any significant difference in the value and vectors of astigmatism between the two devices (95% LoA of 0.99D). A positive and significant correlation between the devices was observed in the mean keratometric astigmatism measurements (R = 0.91, *p* < 0.01). This finding is consistent with the Zhang et al. report, in which 90 eyes (90 subjects) were assessed, and inter-device agreement was noticed in the astigmatism magnitude, cardinal component and oblique component between the devices [16]. Similar to our study, they noticed a positive and significant correlation between the devices (R = 0.881, *p* < 0.0001). Similarly, Huang et al. found the Placido topography (OphthaTop^®^) and Pentacam HR^®^ in complete agreement in the analysis of corneal astigmatism concerning J0 and J45 [15]. Therefore, despite the paucity of literature comparing Pentacam HR^®^ and iTrace^®^, there seems to be a good agreement between the Scheimpflug camera and Placido-based instruments for keratometric measurements on our study and the previous ones.

Pentacam HR^®^ measures both anterior and posterior corneal aberrations; however, the iTrace^®^ device only measures the anterior corneal aberrations. Therefore, only anterior surface HOAs were considered from Pentacam HR^®^ in our study. The iTrace^®^ device has demonstrated a high level of repeatability for measuring corneal aberrations [4]. Although Pentacam^®^ (older model) showed poor repeatability and reproducibility of anterior corneal wavefront aberration [17], the Pentacam HR^®^ was found to report highly precise aberrations in terms of repeatability and reproducibility [18]. The anterior corneal HOAs are the main contributors to calculating total corneal aberrations, based on the published data [19]. Atchison et al. studied 56 eyes of healthy individuals with Pentacam HR^®^ and iTrace^®^ to assess the anterior and posterior corneal aberrations as well as lenticular and total HOA [19]. They noticed that anterior corneal aberrations were approximately three times higher than the posterior corneal aberrations. In our study, anterior corneal Z40 measured with Pentacam HR^®^ was remarkably higher than that of iTrace^®^ (Table 1) with a trend towards positive Z40, compared to iTrace^®^ with a trend towards negative Z40. We noticed a significant difference between the devices with no significant correlations between the Z40 measurements. We could not find any previous study comparing corneal HOA between Pentacam HR^®^ and iTrace^®^. Heidari et al. conducted a study to understand whether HOA could be used for the early diagnosis of subclinical keratoconus [20]. They compared the data between normal eyes and subclinical keratoconus and keratoconus eyes using Pentacam HR^®^, Sirius^®^ and OPD -Scan III^®^. Anterior corneal spherical aberration measured with Pentacam HR^®^ in normal eyes was 0.246 ± 0.069 μm, while OPD-Scan III^®^ aberrometer measured 0.128 ± 0.196 μm in the same group of individuals. Therefore, the tendency of Pentacam HR^®^ to record a more positive Z40 compared to an aberrometer was demonstrated. Similarly, the corneal Z40 measurement is inconsistent even with different aberrometers. Visser et al. assessed the corneal and total ocular aberrations in 23 individuals using four different aberrometer: the Irx3^®^ (Hartmann-Shack; Imagine Eyes, Orsay, France), Keratron^®^ (Hartmann-Shack; Optikon, Rome, Italy), iTrace^®^ (ray-tracing; Tracey Technologies, Houston, TX, USA), and OPD-Scan^®^ (Automated Retinoscopy; Nidek, Gamagori, Japan). Although the iTrace^®^ device was highly repeatable for the corneal aberration measurement, the defocus, trefoil, and spherical aberration measurements differed significantly between most aberrometers [4]. Likewise, Xu et al. evaluated the agreement between a HartmannShack aberrometer (KR-1 W^®^) and iTrace^®^ aberrometer to measure internal and corneal HOA in 50 eyes (50 individuals). Although the devices showed good repeatability, their results were significantly different, and they could not be used interchangeably [21]. They noticed that for cornea HOAs, iTrace^®^ showed higher values than KR-1 W^®^, except in trefoil.

The significant difference between the devices may be justified by the different measurement techniques utilised by these instruments to detect the Z40. In general, iTrace^®^ topography measurement scans a smaller cornea diameter than Pentacam HR^®^ (Personal communication with Tracey Technologies, Houston, TX, USA). Atchison et al. observed that although both instruments scanned the same corneal diameter, they did not scan the same zone of the cornea [19]. As aberrometers operate at lower illuminances than topographers, the pupil size would be larger. The pupil centre could be more temporal for the aberrometer than for the corneal topographer scanning different zones [22]. However, this difference did not affect the measurement of horizontal and vertical coma between the devices in our study (Table 1). We noticed that Pentacam HR^®^ and iTrace^®^ devices were in complete agreement in magnitude and sign of the vertical coma (Z3 − 1) and horizontal coma (Z3 + 1) aberrations with a statistically significant positive correlation between iTrace^®^ and Pentacam HR^®^ (Table 1). This may be theoretically explained because, unlike Z40, Z3 − 1 and Z3 + 1 are not dependent on the difference between the central and peripheral light beam focal points. Therefore, the slight difference in the scanned pupil zones between the devices, induced by the different illumination levels, should not significantly affect the measurement. In healthy corneas, Z3 − 1 and Z3 + 1 have lower values than Z40, leading to less sensitivity in Z3 − 1 and Z3 + 1 measurements than Z40. Wang et al. assessed the central 6 mm corneal and total HOA in 228 eyes of 134 healthy individuals with no known ocular or corneal pathology [23]. Although the values appeared in a wide range, Z40 was higher (0.280 ± 0.086 μm) than the absolute values of the Z3 – 1 (0.157 ± 0.125 μm) and Z3 + 1 (0.150 ± 0.120 μm) in their study. Similarly, Visser et al. found no significant differences in Z3 – 1 and Z3 + 1 measurements between the four different aberrometers [4]. Although we could not find a study comparing the corneal aberrations between Pentacam HR^®^ and iTrace^®^, our findings were not in agreement with the Tabernero et al. study, who compared corneal aberrations between a Placido-based topographer (Medmont E300^®^; Medmont, Camberwell, Australia) and a Hartmann–Shack-based aberrometer (COAS^®^; Asclepion-Meditec-Zeiss, Jena, Germany) [22]. They observed that the effect of the pupillary shift was manifested for coma corneal aberrations. However, the two sets of aberrations calculated with the two pupil positions were not significantly different.

Consideration and effect of pupil size is an important factor in refractive surgery. A larger pupil will increase the level of optical aberrations, in particular spherical aberrations [24]. Wang et al. showed that not all Zernike polynomial coefficients increased for an equal increase in pupil size. Coma aberrations increased less with pupil dilatation, whereas spherical aberration showed only a slight increase from 5–6 mm pupil size [25]. We noticed a significant difference between the devices in pupil measurement. Although the measurements took place in a mesopic room condition for both devices, the devices used different luminance in their measurement. Corneal topography is performed under photopic conditions because of the high luminance of the Placido ring target placed near the eye. In contrast, a small target with low luminance is used in the aberrometer [22]. In a study on the eyes of healthy 20 to 40-year-old subjects, the average pupil size measured by Pentacam HR^®^ was 3.22 ± 0.52 mm [26]. Tabernero et al. noticed the average pupil diameter of 6.4 ± 0.7mm with the Hartmann–Shack-based aberrometer and 3.6 ± 0.6 mm with a corneal topographer [22]. Their results are vis-à-vis to our findings, with an average pupil size of 4.97 ± 1.06 mm with iTrace^®^ and 3.08 ± 0.69 mm with Pentacam HR^®^ (*p* < 0.01).

The limitation of our study is that we did not include eyes with high refractive errors. The same examiner made both sets of measurements, and was therefore not blind to the other device’s measurement. Finally, we did not conduct repeatability and reproducibility tests on the devices, as they are already published with Pentacam HR^®^ [4,18] and the iTrace^®^ [11,21]. However, the strength of our study is that it is the first study on the comparison of pupil size, aberrometry and keratometric measurements between Pentacam HR^®^ and iTrace^®^.

## 5. Conclusions

We concluded that these different devices agreed on measurements of the magnitude and angle of steep and flat meridians, horizontal and oblique astigmatism, and horizontal and vertical coma corneal aberrations. However, the devices produced different pupil size measurements and fourth-order spherical aberration results. Therefore, these instruments cannot be used interchangeably in refractive surgery.

## Figures and Tables

**Table 1:** Mean values measured by iTrace^®^ and Pentacam HR^®^ instruments.

	iTrace^®^ (Mean ± SD) (95% Confidence Interval)	Pentacam HR^®^ (Mean ± SD) (95% Confidence Interval)	Difference (Mean ± SD)	*p* Value *	Pearson Correlation (*p* Value)	Limits of Agreement
Pupil Size (mm)	4.97 ± 1.06 (4.87, 5.07)	3.08 ± 0.69 (3.01, 3.14)	1.94 ± 0.79	<0.01	0.54 (*p* < 0.01)	0.40, 3.48
Z_4_^0^ (µm)	−0.03 ± 0.05 (−0.03, −0.03)	0.30 ± 0.11 (0.29, 0.31)	−0.32 ± 0.11	<0.01	−0.12 (*p* = 0.22)	−0.53, −0.11
Z_3_^−1^ (µm)	0.00 ± 0.05 (−0.01, 0.00)	−0.01 ± 0.25 (−0.04, 0.01)	0.01 ± 0.27	0.78	−0.38 (*p* < 0.01)	−0.53, 0.54
Z_3_^+1^ (µm)	0.01 ± 0.03 (0.01, 0.02)	0.03 ± 0.21 (0.01, 0.05)	−0.03 ± 0.21	0.39	−0.60 (*p* < 0.01)	−0.44, 0.38
J0 (D)	−0.01 ± 0.40 (−0.04, 0.03)	−0.04 ± 0.40 (−0.08, 0.00)	0.03 ± 0.57	0.57	−0.01 (*p* = 0.93)	−1.08, 1.14
J45 (D)	0.06 ± 0.37 (0.03, 0.10)	−0.03 ± 0.38 (−0.06, 0.01)	0.09 ± 0.59	0.09	−0.24 (*p* = 0.02)	−1.07, 1.25
Steepest Keratometry (D)	44.33 ± 2.00 (44.14, 44.52)	44.26 ± 1.86 (44.08, 44.44)	0.10 ± 0.35	0.81	0.97 (*p* < 0.01)	−0.60, 0.79
Steepest meridian (degrees)	93.80 ± 38.41 (90.14, 97.46)	90.11 ± 37.67 (86.51, 93.70)	2.44 ± 31.70	0.49	0.60 (*p* < 0.01)	−59.69, 64.58
Flattest Keratometry (D)	43.40 ± 1.94 (43.22, 43.59)	43.35 ± 1.84 (43.17, 43.53)	0.09 ± 0.32	0.84	0.97 (*p* < 0.01)	−0.53, 0.71
Flattest meridian (D)	78.10 ± 67.43 (71.67, 84.53)	80.12 ± 70.54 (73.39, 86.84)	−2.02 ± 39.23	0.84	0.84 (*p* < 0.01)	−78.92, 74.88
Mean Keratometry (D)	43.87 ± 1.95 (43.68, 44.05)	43.81 ± 1.82 (43.63, 43.98)	0.09 ± 0.31	0.82	0.97 (*p* < 0.01)	−0.51, 0.70
Mean meridian (degrees)	85.95 ± 32.31 (82.87, 89.03)	85.11 ± 32.61 (81.90, 88.32)	1.43 ± 33.61	0.86	0.76 (*p* < 0.01)	−41.86, 44.72
Mean keratometric astigmatism (D)	−0.91 ± 0.59 (−0.97, −0.86)	−0.90 ± 0.61 (−0.96, −0.84)	−0.01 ± 0.26	0.89	0.91 (*p* < 0.01)	−0.52, 0.49

SD, standard deviation; diopters; mm, millimetres; * paired *t* test.

## Data Availability

Not applicable.
